# Head-to-head comparison of CAMPYAIR aerobic culture medium versus standard microaerophilic culture for *Campylobacter* isolation from clinical samples

**DOI:** 10.3389/fcimb.2023.1153693

**Published:** 2023-06-13

**Authors:** Arturo Levican, Carmen Varela, Lorena Porte, Thomas Weitzel, Isabel Briceño, Francisco Guerra, Benjamín Mena, Arthur Hinton

**Affiliations:** ^1^ Tecnología Médica, Facultad de Ciencias, Pontificia Universidad Católica de Valparaíso, Valparaíso, Chile; ^2^ Laboratorio Clínico, Clínica Alemana, Facultad de Medicina Clínica Alemana, Universidad del Desarrollo, Santiago, Chile; ^3^ Instituto de Ciencias e Innovación en Medicina (ICIM), Facultad de Medicina Clínica Alemana, Universidad del Desarrollo, Santiago, Chile; ^4^ Laboratorio Clínico, Hospital Naval Almirante Nef, Viña del Mar, Chile; ^5^ Poultry Microbiological Safety and Processing Research Unit, U.S. National Poultry Research Center, Agricultural Research Service, Athens, GA, United States

**Keywords:** *Campylobacter*, CAMPYAIR, aerobic cultivation, clinical setting, low and middle income countries

## Abstract

*Campylobacter* spp. are considered the most frequent cause of acute gastroenteritis worldwide. However, outside high-income countries, its burden is poorly understood. Limited published data suggest that *Campylobacter* prevalence in low- and middle-income countries is high, but their reservoirs and age distribution are different. Culturing *Campylobacter* is expensive due to laboratory equipment and supplies needed to grow the bacterium (e.g., selective culture media, microaerophilic atmosphere, and a 42°C incubator). These requirements limit the diagnostic capacity of clinical laboratories in many resource-poor regions, leading to significant underdiagnosis and underreporting of isolation of the pathogen. CAMPYAIR, a newly developed selective differential medium, permits *Campylobacter* isolation without the need for microaerophilic incubation. The medium is supplemented with antibiotics to allow *Campylobacter* isolation in complex matrices such as human feces. The present study aims to evaluate the ability of the medium to recover *Campylobacter* from routine clinical samples. A total of 191 human stool samples were used to compare the ability of CAMPYAIR (aerobic incubation) and a commercial *Campylobacter* medium (CASA, microaerophilic incubation) to recover *Campylobacter*. All *Campylobacter* isolates were then identified by MALDI-TOF MS. CAMPYAIR showed sensitivity and specificity values of 87.5% (95% CI 47.4%–99.7%) and 100% (95% CI 98%–100%), respectively. The positive predictive value of CAMPYAIR was 100% and its negative predictive value was 99.5% (95% CI 96.7%–99.9%); Kappa Cohen coefficient was 0.93 (95% CI 0.79–1.0). The high diagnostic performance and low technical requirements of the CAMPYAIR medium could permit *Campylobacter* culture in countries with limited resources.

## 1 Introduction

The zoonotic pathogens, *Campylobacter jejuni* and *Campylobacter coli*, are considered the leading causes of acute bacterial gastroenteritis worldwide. During 2016 in the EU, the number of reported confirmed cases of human campylobacteriosis was 246,307, with a notification rate of 66.3 per 100,000 population being the most commonly reported zoonoses, and representing almost 70% of all the reported cases, followed by salmonellosis, yersiniosis, and STEC infections (EFSA and ECDC, 2017). In the United States, a total of 303,520 culture-confirmed campylobacteriosis cases were reported during 2004–2012, with an average annual incidence rate (IR) of 11.4 cases/100,000 persons, ranging by state from 3.1 to 47.6 cases/100,000 persons (Geissler et al., 2017).

*Campylobacter* spp. also causes other symptoms in humans including acute exacerbation of inflammatory bowel disease and acute appendicitis. Furthermore, extra-intestinal infections such as septic thrombophlebitis, bacteremia, endocarditis, neonatal sepsis, pneumonia, bloodstream infections, brain abscesses, and meningitis may also result from *Campylobacter* infections (Igwaran and Okoh, 2019). Moreover, post-infectious complications associated with campylobacteriosis include reactive arthritis, severe demyelinating neuropathy, Guillain–Barré syndrome, and Miller–Fisher syndrome as well as post-infectious irritable bowel syndrome (Scallan et al., 2015; Igwaran and Okoh, 2019).

Although the epidemiology of campylobacteriosis and its long-term sequelae have been well established in developed countries, the impact of campylobacteriosis in medium- and low-income countries remains underestimated mainly due to insensitive and inconsistently applied isolation methods (Platts-Mills et al., 2014; Porte et al., 2016). In developing countries, *Campylobacter* epidemiology might differ compared to developed regions in that it is usually endemic, asymptomatic, and without marked seasonality (Platts-Mills and Kosek, 2014).

*Campylobacter* gastroenteritis is generally self-limiting, and treatments with antibiotics are not generally recommended, except in prolonged cases, systemic infections, or infections in the elderly, very young, immuno-compromised, or pregnant individuals. *Campylobacter* has become increasingly resistant to certain drugs, however, especially to fluoroquinolones, which are widely used for the treatment of farm animals (Fernandez and Perez-Perez, 2016). Therefore, the World Health Organization (WHO) recently promoted studies addressing the incidence and antibiotic resistance of *Campylobacter* spp., especially in low- and middle-income countries (LMIC) (WHO, 2013). To accomplish this goal, the development and evaluation of isolation methods, which are suitable in environments with limited resources, are crucial (WHO, 2013). Since this is hampered by economic and technical requirements, culture methods that do not require the production of artificial microaerophilic atmospheres during incubation would serve as valuable tools to determine the incidence of *Campylobacter* in locations that are unable to afford traditional *Campylobacter* culturing techniques (Hinton, 2013; Hinton, 2016).

The newly developed selective solid chromogenic medium, CAMPYAIR, supports the growth of *C. jejuni* and *C. coli*. Colonies of the pathogen are usually shiny and purple colored with or without metallic sheen after 48–72 h of incubation under aerobic conditions (Levican and Hinton, 2022). The present study aims to evaluate the ability of this novel medium to recover *Campylobacter* from routine clinical fecal samples compared to a commercially available medium.

## 2 Materials and methods

### 2.1 Preparation of the medium

The CAMPYAIR medium was prepared as described by Levican and Hinton (2022) with the following components and concentrations: beef extract [Merck Millipore, USA; Catalog Number (CN) B4888], 50.0 g/L; tryptose (Sigma-Aldrich Co., St. Louis, MO, USA; CN 70937), 10.0 g/L; sodium lactate syrup, 60% w/v (Sigma-Aldrich Co., USA; CN L1375), 3.0 ml/L; soluble starch (Sigma-Aldrich Co., St. Louis, MO, USA; CN S9765), 1.0%; sodium bicarbonate (Merck Millipore, Burlington, MA, USA; CN 1.06329), 1.5 g/L; agar-agar (Liophilchem, Roseto degli Abruzzi, TE, Italy; CN 611001), 15 g/L; sodium deoxycholate (Merck Millipore, Burlington, MA, USA; CN 106504), 0.1%; 2,3,5-triphenyltetrazolium (TTC, Sigma-Aldrich Co., St. Louis, MO, USA; CN T8877), 200 mg/L; and 800 ml of distilled water. The basal medium was prepared by dissolving all components, which were then sterilized by an autoclave at 121°C for 15 min. The autoclaved media was allowed to cool to 55°C then supplemented with 100 ml of filter-sterilized (0.2 μm filter) 1.5% sodium bicarbonate, 100 ml of defibrinated sheep blood, and the antibiotic supplement containing 32 mg of cefoperazone, 10 mg of amphotericin B (CCDA supplement, Lyophilchem, Roseto degli Abruzzi, TE, Italy; CN 81037), and 10 mg of vancomycin hydrochloride (Sigma-Aldrich Co., St. Louis, MO, USA; CNs 75423). The reference strains *C. jejuni* ATCC 33560 and *C. coli* DSM 4689 were used as positive controls for each batch of the medium.

### 2.2 *Campylobacter* isolation

A correlation study was designed to evaluate the agreement between the results obtained by the new medium CAMPYAIR and the CASA™ (Biomerieux, France) medium. The sample size (*n* = 150) was calculated by using the Epi Info program (CDC; https://www.cdc.gov/epiinfo/esp/es_index.html) with a confidence interval of 99%, 5% of error, and an expected prevalence of 6%, based on a previous data from the same clinical setting (Porte et al., 2016). Fecal samples were obtained consecutively at the Clinical Laboratory of Clínica Alemana, Santiago, Chile. To compare *Campylobacter* isolation, the samples were cultured on CASA™ agar plates (Biomerieux, France) and incubated for 48 h at 42°C under microaerophilic atmospheres (i.e., 5%–10% O_2_, 5%–10% CO_2_, and 80%–90% N_2_) into an anaerobic jar (Anaerocult C, Merck Millipore, USA). Next, the samples were also cultured in parallel on CAMPYAIR agar plates, which were sealed with plastic tape and then incubated for 48–72 h at 42°C under aerobic conditions. During the trials, the CAMPYAIR technique was performed by the laboratory personnel according to the instruction manual without additional instructions or interventions of the research team. Typical *Campylobacter* colonies (i.e., reddish on CASA medium and purple on CAMPYAIR medium) were confirmed by MALDI-TOF mass spectrometry (Vitek MS, Biomérieux).

### 2.3 Statistical analyses

The performance of CAMPYAIR was compared to CASA medium, which previously showed 95% sensitivity and 99.7 negative predictive value (NPV) (Le Bars et al., 2011). Kappa Cohen coefficient was calculated with GraphPad (www.graphpad.com/quickcalcs/kappa2) and used as a measure of agreement. Results were interpreted using the following criteria: 0.01–0.20 slight agreement, 0.21–0.40 fair agreement, 0.41–0.60 moderate agreement, 0.61–0.80 substantial agreement, and 0.81–1.00 almost perfect or perfect agreement. Other performance characteristics such as sensitivity, specificity, negative likelihood ratio, positive predictive value (PPV), negative predictive value (NPV), and accuracy as well as their 95% confidence intervals were calculated using MedCalc (www.medcalc.org/calc/diagnostic_test.php), using culture on CASA medium as reference standard.

## 3 Results

A total of 191 consecutive samples were analyzed in parallel by CASA and CAMPYAIR media, and the ability of the media to recover *Campylobacter* from the samples was compared. *Campylobacter* spp. were detected in 8 (4.2%) samples on CASA medium and in 7 (3.7%) samples on CAMPYAIR medium (**Table 1**). All isolates were confirmed as *C. jejuni*. *Campylobacter* strains grew on CAMPYAIR as large, purple, and partly confluent colonies, while on CASA, colonies were reddish, with variable sizes, but predominantly small pinpoint colonies. An almost perfect agreement was observed between both media, with a 0.93 Kappa Cohen coefficient. Compared to the reference standard, CAMPYAIR had 87.5% (95% CI 47.4–99.7%) sensitivity, 100% (95% CI 98100%) specificity, 100% PPV, and 99.45% NPV (**Table 2**).

**Table 1:** Samples with growth on CAMPYAIR and/or CASA medium.

	CASA medium		CAMPYAIR medium	
Sample no.	Growth semiquantification (colony features)	Bacteria identified	Growth semiquantification (colony features)	Bacteria identified
1	+ (reddish pinpoint)	*C. jejuni*	No growth of *Campylobacter*	No growth
39	+ (reddish pinpoint)	*C. jejuni*	+++ (purple, large, and partly confluent)	*C. jejuni*
72	+ (reddish pinpoint)	*C. jejuni*	+ (purple, large, and partly confluent)	*C. jejuni*
73	+++ (reddish pinpoint)	*C. jejuni*	+++ (purple, large, and partly confluent colonies)	*C. jejuni*
74	+++ (reddish pinpoint)	*C. jejuni*	+++ (purple, large, and partly confluent)	*C. jejuni*
84	No growth	NA	NCCO	*Lactobacillus* spp.*
93	No growth	NA	NCCO	*Lactobacillus* spp.*
94	No growth	NA	NCCO	*Escherichia coli*
106	+++ (reddish pinpoint)	*C. jejuni*	+++ (purple, large, and partly confluent)	*C. jejuni*
121	No growth	NA	NCCO	*Escherichia coli*
131	NCCO	*Salmonella* gr. C	NCCO	*Salmonella* gr. C
147	NCCO	*E. coli*	NCCO	*E. coli*
169	+++ (reddish medium-sized)	*C. jejuni*	+++ (purple, large, and partly confluent)	*C. jejuni*
173	No growth	NA	NCCO	*Lactobacillus* spp.*
176	NCCO	*E. coli*	NCCO	*E. coli*
185	No growth	NA	NCCO	*E. coli*
191	+++ (reddish medium-sized)	*C. jejuni*	+++ (purple, large, and partly confluent)	*C. jejuni*

Growth semiquantification: +, low (only one quadrant); ++, regular (two quadrants); +++, abundant (three or four quadrant). NCCO, No characteristic colonies of Campylobacter observed; *Lactobacillus casei/paracasei/rhamnosus.

**Table 2:** Diagnostic performance of CAMPYAIR medium compared to CASA medium in routine stool samples (*n* = 191).

Parameter	Value	95% CI
Sensitivity	87.5%	47.4%–99.7%
Specificity	100%	98%–100%
Negative likelihood ratio	0.12	0.02–0.78
Positive predictive value	100%	
Negative predictive value	99.5%	96.7%–99.9%
Accuracy	99.5%	97.1%–100%

Growth of non-*Campylobacter* bacterial isolates was observed in nine samples (4.7%, 95% CI 2.5–8.7) on CAMPYAIR medium and in three samples (1.6%, 95% CI 0.5–4.5) on CASA medium. None of these isolates exhibited typical *Campylobacter* morphology. These isolates recovered from both media were identified by MALDI-TOF MS as mostly *Enterobacterales*, which are rod-shaped, Gram-negative, non-spore-forming, facultative anaerobes. Additionally, *Lactobacillus* spp., rod-shaped, Gram-positive, aerotolerant anaerobes or microaerophilic bacteria were also recovered on CAMPYAIR medium (**Table 1**).

## 4 Discussion

To validate a novel simplified *Campylobacter* culture technique, the CAMPYAIR medium protocol was compared head-to-head to a standard culture medium for the ability to recover *Campylobacter* from clinical stool samples. Results indicated that CAMPYAIR had a 0.93 Kappa Cohen coefficient. The new medium also showed high agreement with the traditional, reference method, exhibiting a sensitivity of 87.5% and an NPV of 99.45%, which is in accordance with a previous study (Le Bars et al., 2011). Although there was one was false-negative sample recovered on CAMPYAIR medium, a better growth of *Campylobacter* was observed on this medium with larger colonies being produced. The only sample that was positive by CASA™ agar and negative by CAMPYAIR was the first one included in this study. Therefore, the possibility that this disagreement may be due to the lack of experience of the technician who performed this analysis at the beginning of the study cannot be ruled out.

*C. jejuni* was the only *Campylobacter* species isolated from the stool samples, but *C. coli* showed the same morphological characteristics when grown on CAMPYAIR in previous tests (Levican and Hinton, 2022). *Campylobacter* culture has shown a poor sensitivity compared to molecular and immunological detection, and this poor sensitivity has been explained by the loss of *Campylobacter* viability in clinical specimens stored in transport medium, the number of bacteria present in the sample, and the fact that the typical laboratory culture methods are optimized for *C. jejuni* and *C. coli* and are not set up to detect additional pathogenic *Campylobacter* species like *C. lari* and *C. upsaliensis* (Buss et al., 2019). However, the World Health Organization (WHO, 2013) has promoted studies addressing incidence and antibiotic resistance of *Campylobacter* spp., especially in LMIC, by the development and evaluation of isolation methods by culturing, which are suitable in environments with limited resources. Moreover, it has been stated that the uptake of molecular and immunological detection threatens the utility of the surveillance systems, and their incorporation should be done strategically to maintain and improve surveillance for antimicrobial resistance, outbreaks, and the role of various *Campylobacter* species in human illness (Geissler et al., 2017). In this line, future studies aimed to assess the performance of the medium CAMPYAIR in comparison to non-cultural methods and the growth of other species different to *C. jejuni* and *C. coli* are warranted.

Although both media compared in the present study were able to inhibit the growth of most non-*Campylobacter* bacteria from intestinal flora, CAMPYAIR medium did allow the growth isolates of *Enterobacterales and Lactobacillus*; these last isolates were probably related to probiotics prescription to the patients. However, those bacteria showed their typical morphology on blood agar, i.e., large, smooth, shiny, circular, and raised white colonies for *Enterobacterales*, and small to medium gray colonies that were alpha or non-hemolytic for *Lactobacillus*. In contrast, *Campylobacter* produced colonies with a very different morphology (large, purple, and partly confluent colonies, with or without metallic sheen). In a previous study, Le Bars et al. (2011) have also reported non-specific growth of non-fermenters Gram-negative bacteria and yeasts in 24 of 260 samples (9.2%) on CASA™ medium, while in the present study, less growth of non-*Campylobacter* bacterial isolates was observed, i.e., 4.7% and 1.6% on CAMPYAIR and CASA media, respectively. The selectiveness of CAMPYAIR requires further studies with higher sample numbers and different settings. However, due to the chromogenic properties of CAMPYAIR, *Campylobacter* colonies were easy to differentiate, which is crucial for routine microbiological diagnosis.

In this study, the typical *Campylobacter* colonies on CAMPYAIR obtained after 48–72 h of incubation under aerobic conditions were confirmed by MALDI-TOF mass spectrometry (Vitek MS, Biomérieux). However, in laboratories with limited resources, the isolates can be identified by submitting them to Gram staining, oxidase and hippuricase determination, and motility observation. The isolates of *Campylobacter* spp. are Gram-negative bacilli, curved, S or seagull shaped, positive for oxidase and motility. Moreover, those isolates that are positive for hippuricase can be certainly identified as *C. jejuni*, which account for 90% or more of the cases (Levican et al., 2019).

## 5 Conclusion

The novel CAMPYAIR medium had a very high agreement with the standard technique based on a commercial *Campylobacter* medium on the ability to isolate *Campylobacter* from fecal samples. CAMPYAIR showed a high degree of performance for the selective isolation of *Campylobacter* spp. from stool samples without the need of incubating containers under microaerophilic atmospheres. Because of these lower technical requirements, CAMPYAIR could be useful to implement *Campylobacter* culture in countries with limited resources.

## Data availability statement

The original contributions presented in the study are included in the article/supplementary material. Further inquiries can be directed to the corresponding author.

## Ethics statement

Ethical review and approval was not required for the study on human participants in accordance with the local legislation and institutional requirements. Written informed consent from the participants’ legal guardian/next of kin was not required to participate in this study in accordance with the national legislation and the institutional requirements.

## Author contributions

Conceptualization: AL, AH, LP, IB, and TW. Methodology: AL, CV, FG, and BM. Formal analysis: AL, AH, CV, and LP. Writing—original draft preparation: AL. Writing—review and editing: AL, AH, LP, IB, and TW. Funding acquisition: AL. All authors contributed to the article and approved the submitted version.
